# A Long-Range Foresight for the Medical Application of Apoptosis Specifically Induced by Dd-MRP4, *Dictyostelium* Mitochondrial Ribosomal Protein S4, to Cancer Therapy

**DOI:** 10.3390/biom5010113

**Published:** 2015-02-10

**Authors:** Yasuo Maeda

**Affiliations:** Department of Developmental Biology and Neurosciences, Graduate School of Life Sciences, Tohoku University, Aoba, Sendai 980-8578, Japan; E-Mail: kjygy352@ybb.ne.jp

**Keywords:** apoptosis, cell differentiation, cancer therapy, Dd-MRP4 (*Dictyostelium* mitochondrial ribosomal protein S4), microRNA (miRNA), mitochondria, *Dictyostelim discoideum*, human cancer cells

## Abstract

Apoptosis (programmed cell death) is regarded as ultimate differentiation of the cell. We have recently demonstrated that a targeted delivery of Dd-MRP4 (*Dictyostelium* mitochondrial ribosomal protein S4) suppresses specifically the proliferation of the human cancer cells, by inducing their apoptotic cell death (Chida *et al.*, 2014, doi:10.1186/1475-2867-14-56). This amazing fact was discovered, simply based on the finding that Dd-MRP4 expression is absolutely required for transition of *Dictyostelium* cells from growth to differentiation (Chida *et al.*, 2008, doi:10.1186/1471-2156-9-25; Maeda *et al.*, 2013, doi:10.3390/biom3040943). Dd-MRP4 protein has quite unique structural characters, in that it is highly basic (pI: about 11.5) and interestingly has several nuclear-localization signals within the molecule. In this review, we introduce briefly the efficacy of several apoptosis-inducing substances reported thus far for cancer therapy, and speculate the possible mechanisms, by which apoptosis is specifically induced by Dd-MRP4, on the basis of its structural uniqueness. We also discuss several issues to be solved for the medical application of ectopically expressed Dd-MRP4 in human cancer cells.

## 1. Introduction

Apoptosis is a process of programmed cell death that covers important functions in a wide range of developmental processes such as morphogenesis, and serves as a major defense mechanism to remove potentially dangerous cells like cancer cells. A significant number of ribosomal proteins are known to control the cell cycle and apoptosis through their expression levels. For example, (1) abnormal expression of human RPL7 and RPL13a interfere with cell cycle progression and induce apoptosis, interacting with Mdm2, a ubiquitin ligase that checks on p53 levels; (2) Many eukaryotic ribosomal proteins (S7, S19, L5, L22 and L23) are involved in p53-mediated apoptosis; (3) in humans, RPS3 induces caspase-dependent apoptosis, and some of ribosomal proteons involved in apoptosis are over-expressed in cancer cells (reviewed in [1]). Also, overexpression of human RPS27L promotes p53-induced apoptosis in several cancer cell lines, while siRNA silencing of RPS27L suppresses apoptosis [2]. Recently, it has been demonstrated that yeast cells heterogenously expressing human RPL9 (hRPL9) are resistant to the growth suppressive and lethal effects of externally added copper, while that overexpression of the yeast RPL9 orthologues, RPL9A and RPL9B, causes reduced cell growth [3].

Mitochondria exert a central role in many pathways leading to programmed cell death, and several apoptosis-inducing substances have been reported (Table 1) [4,5,6,7,8,9,10,11,12]. Although the precise mechanisms underlying the role in apoptosis remain to be elucidated and seem to be somewhat different from one species to another, it is mainly controlled by mitochondrial proteins. In mammals, activation of caspases (cysteine proteases that are the main performer of apoptosis) is under the tight control of the Bcl-2 family proteins that primarily act by regulating the release of caspase activators from mitochondria as the central administrator of apoptosis (Table 1). As the case of AIF (apoptosis inducing factor), however, there seems to be a caspase-independent pathway for apoptosis [12].

**Table 1:** Summary of main substances with apoptotic anti-cancer ability.

Substances	Action Mechanisms	References
Dd-MRP4 (*Dictyostelium* mitochondrial ribosomal protein S4 derived from *D. discoideum*)	unknown	[4]
p53 (tumor suppressor)	cytochome c release through activation of proapoptotic Bax (Bcl-2-associated X protein) and Bak (BRI1-associated receptor kinase proteins to drive MOMP)	[5]
Apoptin (a protein of 121amino acids encoded by chicken anemia virus (CAV))	activation of caspse-3 and -9, but not caspase-8	[6]
Cisplatin ((SP-4-2)-diammine-dichloroplatinum; CDDP)	caspase activation	[7]
Oleanonic acid (OA) or Ulsolic acid (UA)	elevation of caspase-3 and -8 activities	[8]
Vitamin E (σ-tocotrienol) or Vitamin E analog (α-TEA)	activation of c-Jun N-terminal kinase (JNK)-mediated apoptosis	[9]
Artemisinin (3R,5aS,6R,8aS,9R,12S,12aR)-octahydro-3,6,9- trimethyl-3,12-epoxy-12H-pyrano [4,3-j]-1,2-benzodioxepin-10(3H)-one; originally derived from *Artemisia annua*)	unknown	[10,11]
Apoptosis-inducing factor (AIF: mitochondrion-localized flavoprotein with NADH oxidase)	caspase-independent apoptosis (possibly induced by elevation of mitochondrial membrane permeability?)	[12]

S29 ribosomal protein (RPS29) has been shown to induce mitochondrial-mediated apoptosis of the human laryngeal carcinoma cell line (Hep2 cells) through the activation of p38 MAPK and JNK signaling [13]. Also, some studies have reported several mitochondrial ribosomal proteins (MRPs) as apoptosis-inducing factors, including the death-associated proteins DAP3 and PDCD9 [14,15]. For example, mitochondrial ribosomal protein L41 (MRPL41) inhibits the growth of cancer cells in nude mice, by induction of p53-induced mitochondrion-dependent apoptosis [16]. High expression of the X-linked ribosomal protein S4 (RPS4X); encoded by human sex-chromosome X), which is implicated for cellular translation and proliferation, is also involved in less aggressive ovarian tumors [17]. Justly, the strict “specificity” to cancer cells should be absolutely guaranteed for the medical application of apoptotic cell death. In this connection, the specificity of apoptosis-inducing substances thus far reported is not well inspected and remains to be extensively tested.

We have previously demonstrated using a *Dictyostelium* model system that the *Dictyostelium* mitochondrial ribosomal protein S4 (*Dd-mrp4*) gene expression is absolutely required for the initiation of cell differentiation: *Dd-mrp4*-null cells, which were prepared using an unique method, fail to initiate differentiation [18,19], while the initial step of cell differentiation is markedly enhanced in *mrp4*^OE^ cells overexpressing the *Dd-mrp4* in the extramitochondrial cytoplasm [20]. In general, growth and differentiation are mutually exclusive but are cooperatively regulated throughout development. Thus, the process of a cell’s switching from growth to differentiation is of great importance not only for the development of organisms but also for malignant transformation, in which this process is reversed. For most cells to terminally differentiate or die by apoptosis, they must exit the cell cycle. In this connection, we have precisely specified a critical checkpoint (growth/differentiation transition: GDT point), from which cells begin to differentiate in response to starvation, in the cell cycle of *Dictyostelium* cells [21,22]. Coupled with this finding, increasing evidence indicates that mitochondria have novel, essential, and multiple functions as the regulatory machinery of the initiation of differentiation, cell-type determination, cell movement and pattern formation, most strikingly evidenced in *Dicyostelium* development [18]. As suggested, we have recently found that an ectopically expressed Dd-MRP4 is able to suppress specifically proliferation of human cancer cells, by means of apoptosis induction [4]. *Dictyostelium* cells are soil microorganism belonging to a kingdom different far from human, which produce many pharmacologically active compounds, including polyketides such as DIFs (differentiation-inducing factors; DIF-1 and DIF-3, *etc.*) [23]. Recently, Kubohara *et al.* [23] have reported that DIF-3 and its derivatives are targeted to mitochondria and suppress growth of tumor cells (HeLa cells), at least partly by possibly functioning as mitochondrial uncouplers to interfere with mitochondrial activity, though the specificity of anti-tumor actions and their involvement in apoptosis remain to be elucidated. 

Nothing is known about the molecular mechanism of Dd-MRP4 action in human cancer cells as well as in *Dictyostelium* cells. In this mini-review, however, we tried to speculate the possible mechanisms by which apoptosis is specifically induced by Dd-MRP4, based on its structural characteristics. Also, we will discuss several issues to be overcome for anti-tumor therapy using ectopically expressed Dd-MRP4 in human cancer cells.

## 2. Structural Characteristics of Dd-MRP4 and Its Possible Mechanisms for Inducing Specifically Apoptosis of Cancer Cells

Mitochondrial ribosomes (MRPs) contain bacteria-type proteins reflecting their endosymbiotic heritage. After reflection on the matter, a subset of these genes is retained within the mitochondrion in eukaryotic cells, but most of mammalian MRPs are products of nuclear genes. Thus these proteins are synthesized in cytoplasmic ribosomes by mitochondria for assembly with the mitochondrially encoded rRNA.

**Figure 1:**
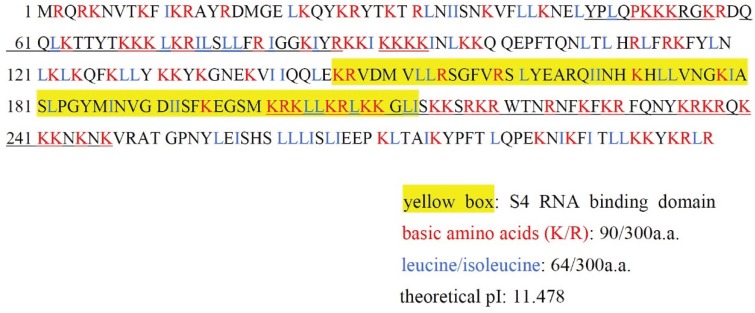
Unique structure of Dd-MRP4 molecule. Based on the presence of S4 RNA binding domain (yellow box), this molecule is categorized into ribosomal protein S4, though its amino acid sequence is markedly different from *Dictyostelium* cytoplasmic ribosomal protein S4 (Dd-RPS4) as a whole. Dd-MRP4 is highly basic (pI: about 11.5) because of the quite high contents of lysine (K) and arginine (R), and interestingly has several nuclear localization signals (underlined regions) within the molecule.

Although *Dictyostelium* is evolutionally far from human, the homology of *Dictysteiulm* cytoplasmic ribosomal protein S4 (Dd-RPS4: 267 amino acids) and human choromosome X-linked cytoplasmic ribosomal protein S4 (Hs-RPS4X; 263 amino acids) is considerably high (66% identity, 92% similarity in the amino acid sequence). In contrast, Dd-MRP4 differs widely from Dd-RPS4 and Hs-RPS4X. Here it is of interest to note that Dd-MRP4 differs considerably from Dd-RPS4 in structure and function, and surprisingly that it has several nuclear localization signals within the molecule (Figure 1; underlined parts). Actually, Dd-MRP4 overexpressed in the cytoplasm of *Dictyostelium* cells has been confirmed to be preferentially transferred to the nucleus [24]. The reason why Dd-MRP4 can be encoded by mitochondrial genome itself in the cytoplasm of *Dictyostelium* cells is mysterious and remains to be elucidated as a chain of evolutionally amazing and rather unexpected incident, because MRPs are generally encoded by nuclear genome in eukaryotic cells. Dd-MRP4 is highly basic (pI: about 11.5), just like histone, and easily imagined to bind readily with DNA and/or RNA. Homology of Dd-MRP4 to Dd-RPS4 (cytoplasmic *Dictyostelium* ribosomal protein) and HRPS4 (human ribosomal protein) is quite low, as previously described [4], though it has a S4 RNA binding domain that probably mediates binding to RNA.

To know the action mechanism of Dd-MRP4, it is of importance to identify substance(s) with which interacts directly to induce specific apoptosis in cancer cells. The direct targets of Dd-MRP4 molecules may be miRNAs (micro-RNAs), considering from its structural and behavioral characteristics. miRNAs are a class of small regulatory RNAs that are implicated for a wide range of cellular processes including apoptosis and cell-cycle control, by means of post transcriptional gene silencing [25]. Human genome encodes 1000 or more kinds of miRNAs consisting of 22–25 single strand RNAs that guide a gene silencing complex to an mRNA by complementary base paring, thus resulting in silencing the gene either translational repression or degradation of the mRNA. A balance between cell proliferation and apoptosis is often disordered in tumors, thus resulting in uncontrolled growth of cancer cells. The recent findings of miRNAs have gained an exciting field of medical research including cancer therapy. Various pathway-specific and cancer-specific miRNAs have been identified during the past several years, suggesting miRNA-mediated regulation of apoptosis in cancers and its future therapeutic applications. In fact, several lines of studies have indicated that some miRNAs serve as oncogenes, and that targeting specific oncogenic miRNAs have therapeutic potentials because of their cancer and pathway specificity [26,27,28]. The effective utilization of antisense-oligonucleotides prepared against oncogenic miRNAs has also been noticed for early medical checkup as promising biomarkers of cancer-type determination. 

The proposed prediction that the direct targets of Dd-MRP4 are oncogenic miRNAs is quite adventurous, because its supporting evidence has not been obtained until now. In such a situation, however, this must be of worth to be intensively tested in future studies, using appropriate methods, though one is not able to neglect other possibilities about the Dd-MRP4 targets. With respect to *Dictyostelium* miRNAs (17 sequences) consisting of 18–26 nucleotides, they were cloned, characterized and developmentally regulated [29], but their functions remain to be elucidated.

## 3. Conclusions and Prospect

For future therapeutic applications of Dd-MRP4 by selective apoptosis of cancer cells, the “extremely high specificity to cancer cells” of its action is quite critical to avoid any side effects to normally growing or differentiating cells. Regrettably, however, the specificity of the apoptosis-inducing substances summarized in Table 1 has not been well evaluated yet. Accordingly, in order to examine the availability of Dd-MRP4 as a potent inducer of apoptosis specific to cancer cells, there are several research subjects to be solved for the time being. First of all, the most active and shortest regions of *Dd-mrp4* gene, which have extremely high specificity to cancer cell, should be determined. For this, deletion experiments of *Dd-mrp4* gene, using cancer cell lines such as hepatocellular carcinoma (HepG2), will be helpful, because HepG2 cells exhibited the most striking response to heterogeneously expressed *Dd-mrp4* [4]. The protein produced from the most active region of *Dd-mrp4* gene is referred to as “MRP4-MC” for convenience. During the process of groping for the MRP4-MC, one can expect that practically useful MRP4-MC molecules that are capable of inducing specifically apoptosis of cancer cells, without giving any side effects on primary cultured cells, will be found out. Such strict “specificity” must be most critical for clinical cancer therapy. Next, it will be needed to know if MRP4-MC actually functions as a potent cancer-specific apoptosis inducer, using various kinds of cancer cell lines. In addition, the transfection efficiency (targeted delivery) of *mrp4-mc* gene should be outstandingly improved for its ectopic expression in human cells. Provided that these problems were fortunately resolved, the strategies presented here should be promising toward preclinical animal studies and then clinical trial for human cancer therapy. In this case, it should be carefully checked whether or not the immunological rejection reaction occurs when MRP4-MC is ectopically expressed in human cancer cells.

It has been sometimes argued that apoptosis is the preferred pathway for therapeutic tumor killing. That is, are there specific therapeutic advantages to killing cancer cells by apoptosis *vs.* necrosis or autophagy? Also, how significant are miRNAs in regulating in these processes? There are precancerous cells and also cancer cells in their various phases of the cell cycle. Anyway, unless one must target them all at a given time by some means including specific induction of apoptosis, one would not be able to kill all the cancer cells at a given time. In days to come, it seems to be most important to kill specifically CSMs (cancer stem cells), which are characterized by mitochondria with a perinuclear arrangement and a low amount of mtDNA, consume less oxygen and have reduced levels of ATP and ROS (reactive oxygen) [30].
